# Socioeconomic inequalities in tobacco cessation among Indians above 15 years of age from 2009 to 2017: evidence from the Global Adult Tobacco Survey (GATS)

**DOI:** 10.1186/s12889-022-13820-7

**Published:** 2022-07-26

**Authors:** Rufi Shaikh, Nandita Saikia

**Affiliations:** 1 International Institute for Population Sciences (IIPS), Mumbai, India; 2 Department of Public Health and Mortality Studies, International Institute for Population Sciences (IIPS), Mumbai, India

**Keywords:** Tobacco, Cessation, Quit, India, GATS, Socioeconomic

## Abstract

**Background:**

Tobacco is strongly associated with socioeconomic status (SES), however evidence on differences in tobacco cessation by socio-economic attributes remains fragmented, especially in developing countries. The present study aims to estimate socioeconomic inequalities in tobacco cessation among Indian men and women above 15 years of age.

**Methods:**

Two rounds of the Global Adult Tobacco Survey (2009–2010 and 2016–2017), India was used to estimate the association between socioeconomic indicators (wealth index and educational attainment) with tobacco cessation using a multinomial modeling approach.

**Results:**

After adjusting for SES and demographic variables, we found significantly lower odds in tobacco cessation rates among respondents of GATS-2 (2016–2017) compared to GATS-1 (2009–2010). Additionally, huge regional variations in smoking and smokeless tobacco cessation rates were observed. Population belonging to the low wealth-asset score had higher odds of cessation compared to the high asset index. While greater educational attainment was seen to have a positive effect on cessation, the results were insignificant. Individuals belonging to the northeastern geographic region were seen to have the lowest odds of cessation. Though awareness about the health hazards of tobacco increased, cessation declined for both men and women. Quitting smokeless tobacco among men and women was observed to be lower than smoking.

**Conclusion:**

This study is the first to provide national-level evidence on the association between tobacco cessation and socioeconomic attributes among Indians above 15 years of age. Findings suggest the need to scale up tobacco cessation services separately for men and women, and also for smoking and smokeless tobacco forms.

**Supplementary Information:**

The online version contains supplementary material available at 10.1186/s12889-022-13820-7.

## Introduction

Tobacco consumption caused over eight million deaths each year across the globe [1], giving rise to tremendous economic harm in the form of excess health-care costs and lost productivity [2]. 80% of these deaths will occur in low-and-middle-income countries (LMIC). India has over 275 million tobacco users, with 164 million users of smokeless tobacco only, 69 million exclusive smokers, and 42 million using both forms [3]. It was estimated that more than one million deaths every year were attributable to tobacco in India [4, 5]. Hence, tobacco control was identified as an immediate priority to reduce NCDs [6].

Worldwide, tobacco consumption showed a persistent declining trend, largely attributed to increased tobacco control policies [7]. In India, tobacco consumption was found to be highly prevalent among middle-aged adults, the illiterate population, and those residing in the rural areas of the country [8]. Substantial spatial differences in the progression of the tobacco epidemic in India were seen between 1998 and 2016 wherein although the national prevalence of tobacco consumption was seen to decline, it was observed to increase in a few states among both gender [9]. Along with regional, cultural and socio-economic disparity, Patel et al. [10] also found educational differences in tobacco consumption. Inequality in consumption of smoked tobacco was seen to decline from -0.14 to -0.1 while that of smokeless tobacco was observed to increase from -0.16 to -0.2 between 2005 and 2016.

Although national-level tobacco rates have been falling, reductions have been slower amongst the disadvantaged, and inequalities are seen to increase [11]. Additionally, tobacco use is strongly associated with socioeconomic status (SES) [12] and contributes to inequalities in tobacco-attributable mortality and morbidity [13]. Rossouw [14] found tobacco consumption to be concentrated among the poor in China, India, Ghana, and South Africa and stated that the major drivers of inequality include wealth, locality, and gender. These inequalities in tobacco consumption are driven by disparities in both initiation and cessation of tobacco use across different socioeconomic status [15]. Discrepancies by SES are especially worrying, as tobacco prevalence among those with low SES is the highest and declining less rapidly compared with higher SES [16]. SES differences in quit rates and low quit rates among the disadvantaged have also been observed in studies of cessation interventions [11, 15, 17]. In a systematic analysis of 42 studies, Kock et al. [18] found that individual-level interventions can assist disadvantaged smokers with quitting. However, they found no large moderating effects of tailoring for disadvantaged smokers. Another study found that people living in socioeconomically deprived areas or with lower education and/or wealth are reported to be more inclined towards tobacco and less successful in quitting [19]. Analyzing the socioeconomic determinants of tobacco cessation helps to explain how policies aiming to reduce tobacco are translated in terms of distribution and social benefits among different socioeconomic groups. Existing evidence on differences in quitting tobacco by SES along different dimensions—such as income, wealth, education, occupation, and residence—mostly pertains to high-income countries [15]. These studies have consistently found that lower SES is predictive of a lower probability of tobacco cessation. However, research on whether those with lower SES have a varying likelihood of successfully quitting tobacco compared to those with higher SES remains fragmented in LMICs.

In India, three studies [15, 20, 21] have examined the relationship between tobacco quitting and socioeconomic status. Nargis et al. [15] did not find any significant association between SES and national tobacco cessation whereas Srivastava [20] found a significant positive association between male gender, increased educational attainment, and higher wealth quintile for those who attempted to quit and who were successful in quitting tobacco during 2010. Corsi et al. [21] found higher quit rates among women than men and a positive association between smoking quit rates and SES in rural Andhra Pradesh. Given the mixed findings, more studies are required to systematically examine the interdependence between SES indicators and quitting outcomes in India. To address this gap, the present study aims to examine the socio-economic inequalities in tobacco cessation behavior among Indian men and women, where substantial progress has been made in implementing tobacco control policies over the last decades [15]. Such comparisons of tobacco cessation across the socioeconomic hierarchy will not only provide valuable information for future policy formulations regarding tobacco cessation programs but will also provide a benchmark to gauge whether national policies are progressing toward equity in health.

## Methods

### Data

The present study used The Global Adult Tobacco Survey (GATS) for India conducted during 2009–2010 and 2016–2017 [3, 22]. GATS is the global standard for systematically monitoring tobacco use and tracking key tobacco control indicators. It was carried out in all 29 states and 2 union territories (Chandigarh and Puducherry) of the country. The major objective of the survey was to obtain estimates on the prevalence of tobacco use; exposure to second-hand smoke; cessation, economics, exposure to media messages, and knowledge, attitude, and perception towards tobacco use. A total of 69,296 interviews were completed during GATS1 (2009–2010) among which 33,767 and 35,529 were males and females respectively. Out of all completed interviews, 41,825 interviews were conducted in rural and 27,471 interviews in urban areas. Similarly, 74,037 individual interviews were completed during GATS2 (2016–17) out of which 33,772 were males and 40,265 were females. Out of all completed interviews, 26,488 and 47,549 interviews were completed in urban and rural areas respectively. The overall response rate was 91.8 percent in GATS1 and 92.9 percent in GATS2.

### Outcome measure

For both survey rounds, the outcome variable considered was successful tobacco smoking and smokeless tobacco cessation during the past 12 months. Information on tobacco use was obtained through survey respondents using a structured questionnaire. Current smokers and smokeless tobacco users were defined as all those respondents who were using any form of tobacco in either smoked or smokeless form at the time of the survey. Smoked tobacco included manufactured cigarettes, hand-rolled bidis, cheers, cigars, hookah, etc. while smokeless tobacco included all types of chewing tobacco. Tobacco cessation was defined as the percentage of ever tobacco users (current and former) who quit tobacco use for the past 12 months at the time of the survey. The following questions were used to define tobacco cessation: 1) During the past 12 months have you tried to stop smoking/ smokeless tobacco and 2) Thinking about the last time you tried to quit, how long did you stop smoking/ smokeless tobacco? Respondents who answered "yes" to the first question and reported 12 months of quitting tobacco were used to obtain the proportion of tobacco cessation among Indian adults. Due to the diverse use and dynamics involved in using tobacco products in India, we considered cessation of tobacco smoking and smokeless tobacco separately.

### Demographic and Socio-economic characteristics

The demographic characteristics considered were age and sex. Age was categorized as < 25 years, 25–44 years, 45–64 years, and above 65 years of age. Other demographic characteristics taken into account were place and region of residence.

While defining the socio-economic constructs of a population, often education, occupation, and income level are used interchangeably to measure the socioeconomic position [23]. Among the socioeconomic determinants, social group and religion are usually non-modifiable characteristics (fixed from birth), whereas other determinants–education, occupation, and wealth (a proxy indicator of income)–are achievable or modifiable. We used the education and wealth asset index as proxy markers of the socioeconomic status of the respondent to fulfill the current study objective. Education was categorized as individuals with no formal education, who completed primary, secondary, higher, and above education. To compute the wealth asset index for the data, we used the methodology adopted by the Demographic and Health Survey (DHS) where households were given scores based on the number and kinds of assets they owned, ranging from a television to a bicycle or car, and housing characteristics such as the source of drinking water, toilet facilities, and flooring materials. These scores were derived using the principal component analysis (PCA). National wealth quintiles were compiled by assigning the household score to each usual (de jure) household member, ranking each person in the household population by their score, and then dividing the distribution into five equal categories, each with 20% of the population. For the present study, we categorized the wealth asset index into three categories of a low, medium, and high wealth asset scores where the lowest two quintiles represented individuals belonging to the poorest wealth asset quintile, and the third quintile constituted the population of the middle wealth quintile and the last two represented individuals belonging to the richest wealth quintile.

In addition to the demographic and socioeconomic characteristics, individuals' awareness and perception of the harmful hazards of tobacco use were also obtained. Awareness about the negative health effects of tobacco use was defined as the proportion of individuals noticing information on the dangers of tobacco use in magazines, newspapers, television, radio, billboards, public transport, etc., or cigarettes, bidi, and smokeless tobacco packages. Respondents who answered "yes" to the question "In the last 30 days did you notice any information in newspapers or magazines/ television/ radio/ billboards/ public transport about the dangers of use or then encourages quitting of the tobacco products?” were categorized to be aware of the ill-effects of tobacco use. Similarly, the perception that tobacco has detrimental effects on the body was defined as the percentage of individuals believing that tobacco use causes serious illness, stroke, heart attack, cancers, etc. Respondents who answered “yes” to the following two questions: 1) Based on what you know or believe, does smoking/smokeless tobacco cause serious illness? and 2) Based on what you know or believe does smoking/ smokeless tobacco cause stroke/ lung cancer/ heart attack? were classified as individuals who perceived that tobacco had negative impacts on the body.

### Statistical analysis

Descriptive analysis reporting the study characteristics was weighted using the sampling weights provided by the survey. These weights took into account unequal probabilities of selection that emerged during sampling procedures as well as survey non-response. Bivariate analysis with *P* values was calculated to obtain the distribution of tobacco use and cessation prevalence with socioeconomic indicators (wealth index and education). We merged the two rounds of the GATS survey to analyze the significance of tobacco cessation over time along with individuals' socioeconomic attributes. A multivariate logistic regression model was used to examine the effect of socioeconomic indicators on tobacco cessation over time. Separate models were fitted for tobacco smoking cessation and smokeless tobacco cessation among men and women. Tobacco cessation was modeled as a dichotomous outcome, Pr(y = 1), assumed to be binomially distributed with probability conditional on different demographic and socioeconomic variables by a logit link function.

$$\mathrm{Logit}\lbrack\mathrm p\left(\mathrm Y\right)/\left(1-\mathrm p\left(\mathrm Y\right)\right)\rbrack=\mathrm\beta0+\mathrm\beta1\mathrm X1+\mathrm\beta2\mathrm X2+......+\mathrm\beta\mathrm{pXp}$$

where:

Y: Outcome variable – Tobacco cessation.

Xj: The jth predictor variable.

Βj: The coefficient estimate for the jth predictor variable.

The right side of the equation predicts the log odds of the response variable taking on a value of 1.

All analysis was done using STATA 16.

## Results

Table 1 gives the demographic and socioeconomic characteristics of the study population for GATS 1 and GATS 2. The distribution of men and women and age was approximately similar during 2009–2010 and 2016–2017. 70.7% of the study population live in rural areas during 2009–2010 which decreased to 65.5% in 2016–17. 31% of the population had no formal education during 2009–2010 which reduced to 26.4% in 2016–17. Contrary, 16.3% population had higher and above educational attainment which rose to 22.1% in 2016–2017. Approximately 25% population during 2009–2010 belonged to the low wealth asset tercile and 43% to the high wealth asset index whereas 28% population are classified to have a low wealth asset score compared to 32.5% belonging to the high wealth asset index during 2016–17.

**Table 1:** Sociodemographic and economic characteristics of the study population in GATS 1 (2009–2010) and GATS2 (2016–2017)

Sociodemographic and economic characteristics	Study population			
	**GATS 1 (2009–2010) *N* = 69,296**		**GATS 2 (2016–2017) *N* = 74,037**	
	**%**	**N**	**%**	**N**
**Demographic Characteristics**				
Gender				
Male	51.68	33,767	51.10	33,772
Female	48.32	35,529	48.90	40,265
Age				
< 25	29.50	13,463	26.73	13,329
25–44	42.03	35,020	44.44	35,564
45–64	21.70	16,123	21.73	19,132
65 +	6.76	4,690	7.11	6,012
Residence				
Rural	70.77	41,825	65.51	47,549
Urban	29.23	27,471	34.49	26,488
**SES**				
Educational Attainment				
No formal education	31.00	18,805	26.42	18,473
Primary	24.00	16,303	20.54	16,368
Secondary	28.70	20,185	30.95	22,440
Higher and Above	16.31	13,863	22.08	16,697
Wealth Index				
Low	25.02	24,190	28.41	24,966
Medium	32.23	22,057	39.13	26,310
High	42.75	23,048	32.46	22,761

**Fig. 1:**
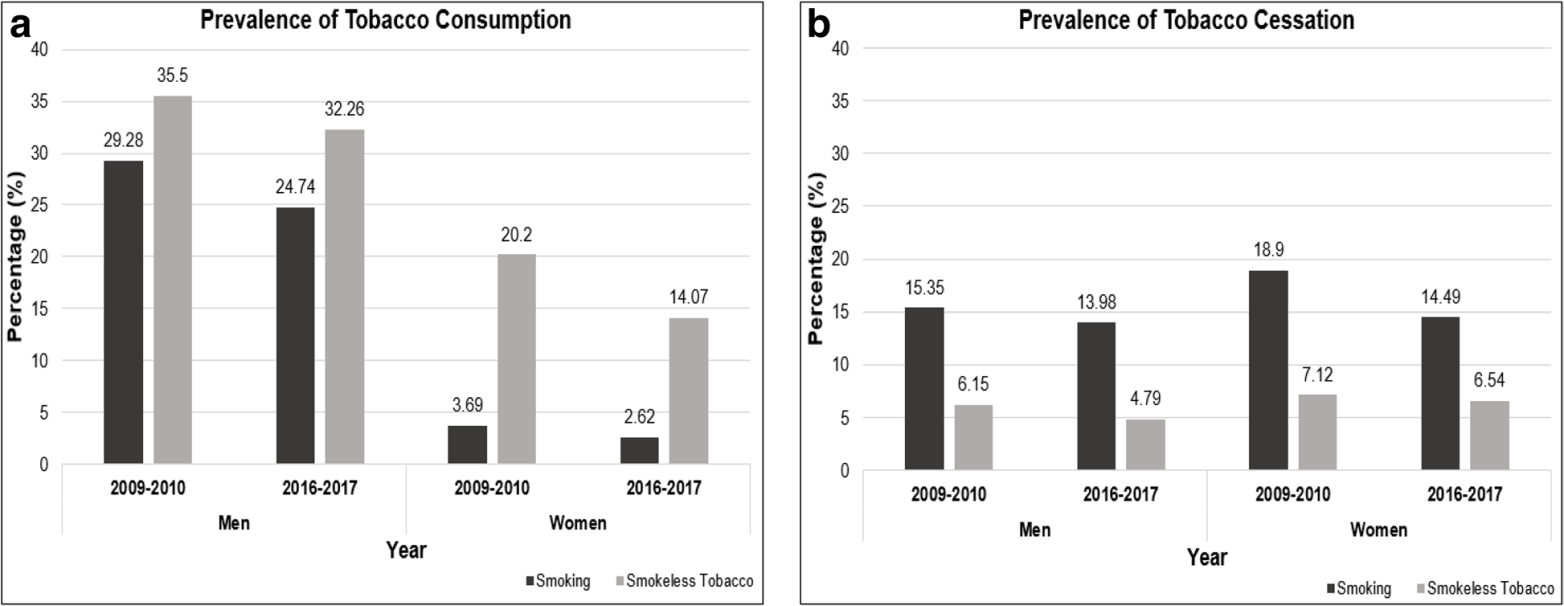
Prevalence of tobacco consumption and cessation among Indian men and women above 15 years of age during 2009–2010 and 2016–2017

Prevalence of tobacco smoking declined (Fig. 1a) by 16% (2009–2010:29.28% to 2016–2017:24.74%) among Indian men and by 29% (2009–2010:3.69% to 2016–2017:2.62%) among Indian women whereas that of smokeless tobacco reduced by 9% (2009–2010:35.50% to 2016–2017:32.26%) among men and 30% (2009–2010:20.20% to 2016–2017:14.07%) among women from 2009 to 2017. Similar to consumption, tobacco cessation rates between 2009 and 2017 (Fig. 1b) among men reduced by 9% for tobacco smoking and 22% for smokeless tobacco. Similarly, quitting rates among women declined by 23% for tobacco smoking and 8% for smokeless tobacco between 2009 and 2017.

**Fig. 2:**
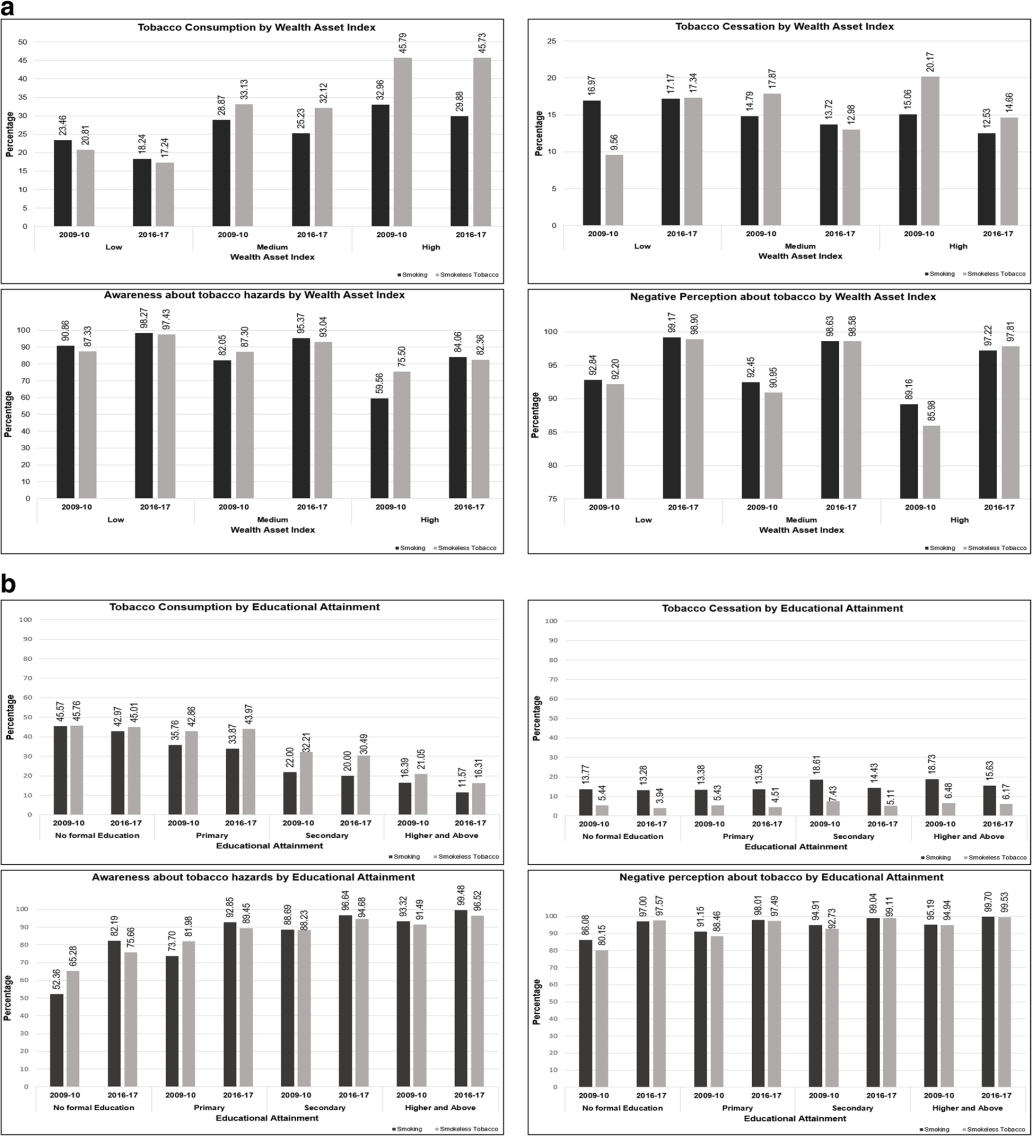
**a** Prevalence of tobacco consumption, cessation, awareness, and perception about hazards of tobacco use among Indian men above 15 years of age by wealth index during 2009–2010 and 2016–2017. **b** Prevalence of tobacco consumption, cessation, awareness, and perception about hazards of tobacco use among Indian men above 15 years of age by educational attainment during 2009–2010 and 2016–2017

Figure 2 represents the prevalence, and cessation along with the knowledge, attitude, and perception of men towards tobacco smoking and smokeless tobacco according to their socioeconomic attributes (wealth index and education attainment) between 2009 and 2017. (Appendix Table 1a and 1b). While tobacco consumption is seen to increase from the low to the high wealth asset tercile (Fig. 2a), cessation is observed to decline as we move up the wealth index score. Tobacco smoking cessation between 2009 to 2017 is seen to decline by 1%, 7%, and 17% among men belonging to low, medium, and high wealth asset index respectively whereas cessation of smokeless tobacco is seen to reduce by 21% among men belonging to the low and high wealth index and by 29% among men from the medium wealth asset score. Though an increase in awareness and perception about the hazards of tobacco use is witnessed among men between 2009 to 2017, it is seen to decline as we move up the wealth asset score during both the survey rounds. With an increase in educational attainment (Fig. 2b) among Indian men, tobacco consumption (both smoked and smokeless tobacco) is observed to decline except for men with secondary level education where the prevalence of smokeless tobacco was seen to increase by 3% between 2009 and 2017. Similarly, tobacco cessation among men with no formal education, primary and higher and above education is seen to reduce from 2009 to 2017, while a 1% increase is witnessed in tobacco smoking cessation among men having completed secondary education. Contrary to wealth asset score, awareness and perception about the negative effects of tobacco smoking and smokeless tobacco on educational attainment are observed to increase with an increase in educational qualification between 2009 and 2017.

**Fig. 3:**
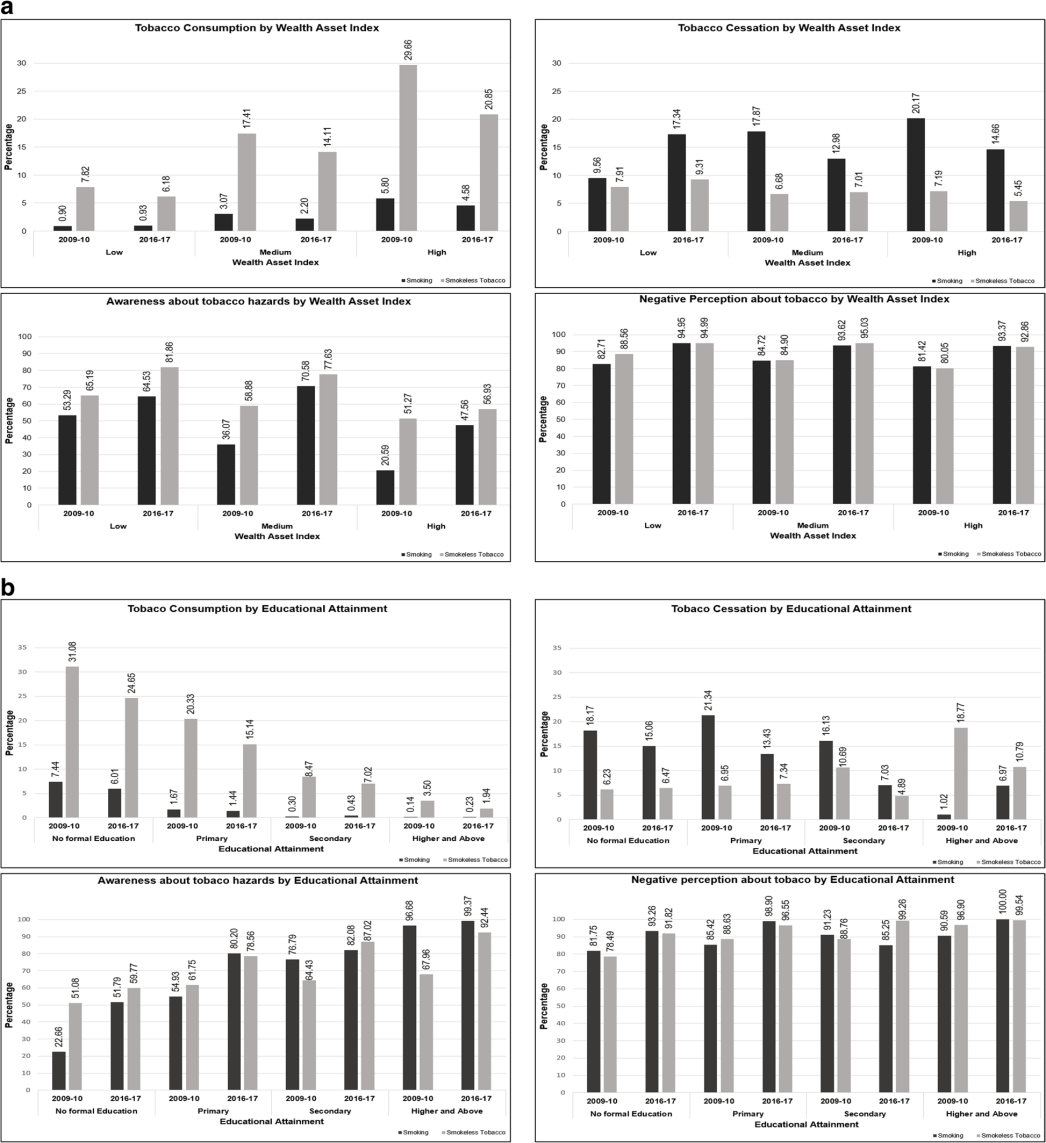
**a** Prevalence of tobacco consumption, cessation, awareness, and perception about hazards of tobacco use among Indian women above 15 years of age by wealth index during 2009–2010 and 2016–2017. **b** Prevalence of tobacco consumption, cessation, awareness, and perception about hazards of tobacco use among Indian women above 15 years of age by educational attainment during 2009–2010 and 2016–2017

Indian women are noticed to have substantially different patterns of tobacco consumption and cessation as compared to men. Women belonging to the low wealth asset score (Fig. 3a) are observed to have a 3% increase in tobacco smoking consumption and 81% increase in smoking cessation between 2009 and 2017 while smoking consumption and cessation among women belonging to the medium and high asset index is witnessed to decline between 2009 to 2017 (Appendix Table 2a). Like men, awareness and perception of hazards of tobacco consumption are witnessed to increase between the two survey rounds and seen to decline with an increase in wealth asset score. While women with no formal and primary education observed reductions in tobacco smoking (Fig. 3b), those with secondary and higher education saw a 43% and 64% increase in tobacco smoking between 2009 and 2017 (Appendix Table 2b). Smoking cessation between 2009 and 2017 is seen to decline among women with no formal, primary, and secondary education, whilst it is seen to increase among women with higher and above educational attainment. Although the use of smokeless tobacco noticed declined, quitting smokeless tobacco is seen to increase by 4% and 6% among women with no formal and primary education respectively, and is witnessed to reduce by 17% and 45% among women with secondary and higher and above educational qualification respectively. Awareness and perception of the negative effects of tobacco consumption are seen to increase substantially with an increase in educational attainment among Indian women.

**Table 2:** Association between socioeconomic status and tobacco cessation among Indian men and women above 15 years of age during 2009–2010 and 2016–2017

Population Characteristics	Tobacco Cessation POR (95% CI)			
	**Smoking**		**Smokeless**	
	**Men**	**Women**	**Men**	**Women**
**SES**				
Wealth Index				
Low ®				
Medium	**0.81 (0.72,0.90)**	0.77 (0.54,1.10)	**0.73 (0.63,0.86)**	0.88 (0.71,1.10)
High	**0.73 (0.65,0.83)**	**0.63 (0.91,0.55)**	**0.65 (0.55,0.78)**	**0.70 (0.56,0.89)**
Educational Attainment				
No formal education®				
Primary	**1.19 (1.06,1.33)**	1.10 (0.79,1.53)	1.12 (0.94,1.34)	1.17 (0.97,1.41)
Secondary	**1.39 (1.23,1.58)**	0.88 (0.53,1.44)	**1.42 (1.19,1.70)**	1.22 (0.97,1.54)
Higher and Above	**1.49 (1.28,1.74)**	0.65(0.26,1.60)	**1.31 (1.05,1.64)**	**1.89 (1.35,2.64)**
**Demographic Characteristics**				
Age				
< 25 ®				
25–44	0.94 (0.80,1.11)	0.81 (0.50,1.32)	1.02 (0.84,1.25)	0.90 (0.72,1.13)
45–64	**1.59 (1.35,1.87)**	0.98 (0.60,1.61)	**1.39 (1.13,1.72)**	**0.73 (0.56,0.94)**
65 +	**3.08 (2.58,3.69)**	1.17 (0.68,1.99)	**2.22 (1.73,2.84)**	0.94 (0.69,1.28)
Residence				
Rural ®				
Urban	0.91 (0.83,1.00)	1.20 (0.89,1.61)	1.05 (0.92,1.20)	1.17 (0.99,1.38)
Region				
North ®				
Central	**1.29 (1.11,1.49)**	**0.62 (0.40,0.96)**	1.04 (0.84,1.27)	**0.53 (0.39,0.72)**
East	**1.23 (1.07,1.41)**	**1.87 (1.32,2.66)**	**0.55 (0.44,0.68)**	**0.74 (0.56,0.97)**
North East	**0.64 (0.57,0.73)**	**0.43 (0.31,0.59)**	**0.64 (0.54,0.78)**	**0.25 (0.19,0.33)**
West	**1.21 (1.01,1.45)**	0.81 (0.42,1.58)	0.99 (0.79,1.24)	**0.57 (0.41,0.79)**
South	**1.79 (1.58,2.02)**	**0.56 (0.33,0.98)**	**1.66 (1.34,2.05)**	**0.68 (0.50,0.92)**
**Knowledge, Attitude, and Practise**				
Awareness				
No ®				
Yes	**0.65 (0.58,0.73)**	**0.65 (0.51,0.84)**	**0.78 (0.66,0.91)**	**0.72 (0.61,0.84)**
Perception				
No ®				
Yes	**2.05 (1.64,2.55)**	**2.19 (1.42,3.38)**	1.16 (0.92,1.47)	**1.52 (1.17,1.97)**
Round				
GATS 1 (2009–2010) ®				
GATS 2 (2016–2017)	0.95 (0.87,1.04)	**0.78 (0.61,1.00)**	**0.83 (0.73,0.93)**	**0.77 (0.66,0.90)**

Values in bold are significant at a 5% level of significance

Association between tobacco cessation and socioeconomic characteristics (educational attainment and wealth index) among Indian men and women between 2009 and 2017 is represented in Table 2. Men and women belonging to the high wealth asset index had comparatively lower odds of tobacco cessation than those from the low wealth asset index. The odds of men belonging to a high wealth index and quitting smoking and smokeless tobacco were 27% and 35% less than their counterparts belonging to a low wealth index respectively. Similarly, women from a high wealth asset index had 37% and 30% less likelihood of tobacco smoking and smokeless tobacco cessation compared to women from a low wealth asset index. Higher education was seen to have a positive effect on tobacco cessation. However, women who had higher educational attainment were found to have a 35% less chance of quitting smoking compared to those women with no formal education. Elderly men above 65 years of age had 3.08 and 2.22 times higher odds of smoking and smokeless tobacco cessation respectively compared to men less than 25 years of age. Similarly, women above 65 years had 1.17 times higher odds of quitting tobacco smoking than women below 25 years of age. Contrary to men, women above 65 years of age had a 6% less chance of quitting smokeless tobacco compared to those below 25 years of age. Smoking cessation was seen to be higher among urban men whereas smokeless cessation was higher among rural men. Cessation of tobacco smoking and smokeless tobacco was higher among urban women compared to their rural counterparts. However, the association between residence and tobacco cessation was found to be insignificant at a 5% level of significance. Distinct regional patterns have been observed in tobacco smoking and smokeless cessation among men and women in India. The odds of quitting tobacco smoking were highest among men in South India compared to North while northeastern men had a lower odds of quitting smoking compared to north Indian men. Similarly, men in South India had 1.66 times higher odds of quitting smokeless tobacco whereas those in East India had 50% less chance of smokeless tobacco cessation compared to men in Northern India. Likewise, women in East and Northeast India had 1.87 times higher odds and 0.43 times lower odds of smoking cessation than women residing in North India. Similarly, women in North India had higher odds of quitting smokeless tobacco compared to other regions. While on one hand, men and women having awareness about the health hazards attributable to tobacco smoking and smokeless tobacco were found to have lower odds of tobacco cessation compared to those not having awareness; on the other hand, perception of the negative effects of tobacco consumption on health was seen to have a significantly higher odds of tobacco cessation among both men and women in India. A decline in cessation rates was found to be significant among women for tobacco smoking and among men and women for smokeless tobacco at a 5% level of significance and was higher for tobacco smoking than smokeless tobacco.

## Discussion

A significant decline in cessation rates for tobacco smoking and smokeless tobacco among men and women was observed in India between 2009 and 2017. Men and women belonging to the low wealth asset score had higher odds of tobacco cessation compared to those from the high asset index. While higher educational attainment was seen to have a positive effect on tobacco cessation, the results were however statistically insignificant. Men and women belonging to the northeastern geographic region were seen to have the lowest odds of tobacco cessation compared to those from the northern region. Though awareness about health hazards due to tobacco increased, cessation was seen to decline for both men and women. Cessation of smokeless tobacco among men and women was observed to be lower than tobacco smoking between 2009 and 2017.

Though India witnessed small declines in the prevalence of tobacco smoking and smokeless tobacco use between 2009–2010 and 2016–2017, a significant reduction in tobacco cessation rates was also observed during the same time. This result is quite different from high-income and other low/middle-income countries where tobacco cessation rates have improved over time [24], and therefore may be a result of the lack of effective tobacco control policies exercised in the country. A decrease in tobacco cessation rates also points to the fact that tobacco control strategies based on the FCTC in India may not be enough to control the use of tobacco in the nation [21].

Our results showed that there have been large socioeconomic inequalities in cessation of tobacco smoking and smokeless tobacco by wealth asset index and education from 2009 to 2017. These differentials were relatively large for tobacco smoking cessation as compared to smokeless tobacco cessation, and were higher among women than men. Indian men and women belonging to a low wealth asset index had higher odds of cessation rates compared to those belonging to the high wealth index score. This might be due to the tobacco control strategies adopted by a country which may account for the socioeconomic gradient of quitting behaviour [15]. For example, in a country that is actively pursuing tobacco tax and price policy, low SES individuals, who are more responsive to tax and price increases, could be more successful in quitting compared to high SES [15]. Findings from our study are in line with observations from developed countries where higher quit ratios were observed among the population with low SES [16, 25].

Individual emancipation and increase in social status have long been associated with increase in tobacco consumption [26] and this can explain the low quit rates for tobacco in India with higher educational attainment. The young urbanized population is often among the first to adopt tobacco [27]. Though Indian women were observed to have higher quit rates compared to men, huge differentials were observed in cessation rates with socioeconomic attributes. Women with higher SES were seen to have low cessation rates. This might be due to the rise in purchasing power and access to tobacco products associated with an increase in SES, peer pressure, attraction towards role models and symbols to be modernized [28] as well as different marketing strategies adopted by the tobacco industry [26]. Results from the study also display that the key drivers of the progression of the tobacco epidemic such as increasing gross domestic product in developing countries (which increases cigarette purchasing power) and increasing educational status (which increases awareness of health risks) have a differential impact on women and men, and are mediated by gendered social, cultural and economic factors [27]. These factors include social norms and restrictions on women’s smoking, as well as shifts in women’s social and economic status. Indian women with no formal education were seen to have higher quit rates compared to those with higher educational attainment. This might be suggestive that women with higher SES have greater purchasing power and are more addicted to the consumption of tobacco than those with lower SES [26, 27]. Addiction is also witnessed to play an important part and is considered to be one of the key drivers along with the socioeconomic status for lower cessation rates [29, 30]. Similar findings were seen in an analysis of smoking initiation and quitting behavior among Irish women [31] which stated that taxation is the most effective strategy in inducing quitting among those with the lowest level of education.

Apart from disparities in tobacco cessation with socioeconomic attributed to wealth asset index and educational attainment, huge regional variations in cessation rates were observed in India. Smoking cessation (Appendix Table 3a) among men during 2016–2017 ranged from 3.92%, 4.43%, and 5.42% in Goa, Meghalaya, and Tripura respectively to 21.82%, 29.36%, and 31.52% in Odisha, Kerala, and Bihar respectively whereas that for women extended from no cessation in Nagaland, Himachal Pradesh, Punjab to 52.64%, 60.85% and 69.18% in Assam, Chhattisgarh, and Odisha respectively. Similarly, cessation of smokeless tobacco (Appendix Table 3b) varied from 0.77% in Tripura to 23.55% in Kerala among men and from no cessation in Haryana and Punjab to 26.48% in Kerala among women. Low cessation rates among the north-eastern Indian states where tobacco use is still increasing in some parts [9] might be due to the high social acceptance of tobacco use and cultural practices. It also points to the fact that the tobacco control policies in these states were less effective in minimizing the use of tobacco products [32].

The variety of forms of tobacco use in India poses a challenge for tobacco control efforts [33]. Inequalities in tobacco smoking and smokeless tobacco cessation by wealth index and educational attainment among men and women were observed in India. Similar findings of divergent socioeconomic patterning by type of tobacco were also observed in a rural Indian setting suggesting that the underlying factors driving the use of each type may vary across socioeconomic strata [21]. India has a long history of implementing tobacco control measures since the 1990's [9]. Cessation rates were higher for tobacco smoking as compared to smokeless tobacco among both men and women. One reason for this difference could be the differential impact of early anti-tobacco measures that focused primarily on tobacco smoking [34]. India introduced a host of anti-tobacco measures after the ratification of the FCTC in 2004, but these measures initially primarily focussed on controlling smoking [35]. Some of these strategies included a ban on sale to minors, point-of-sale advertisements, a ban on sale near educational institutions, a ban on smoking in public places, and the implementation of tobacco packaging health warnings. Although some of these regulations (advertisements and educational institutions' embargoes) applied to smokeless tobacco products as well, the law specifically banned the manufacture and sale of gutka and paan masala (major smokeless tobacco products in India) only in 2013 [34]. Despite these legislations, the use of smokeless tobacco is widely prevalent among the population. Taxation, one of the most effective tobacco control measures, has been very beneficial in reducing smoking prevalence in India [36], especially among the underprivileged sections. However, the smokeless tobacco market has evaded the tax net for far too long. Taxation on cigarettes and other smoked products has continued to rise between 2008 and 2015, but no sizeable increase in taxes on smokeless tobacco products was seen, encouraging their unabated use [37]. Along with national-level tobacco control policies, various awareness campaigns such as the "Quit Tobacco Movement (2008), "Life se Panga Mat Le Yaar (2011), Election Campaign (2014), and “Tambakhu ko Dhishum (2015)” were launched to encourage individuals to stop using tobacco. However, though awareness about the health hazards of tobacco consumption increased among the Indian population, the odds of quitting smoking and smokeless tobacco were statistically lower as compared to those individuals who were not aware of the negative ill-effects of tobacco consumption. It was also seen that awareness about the hazards of tobacco consumption was highest amongst individuals belonging to the lowest socioeconomic status, which is similar to the findings observed by Kankaria et al. [38]. High cessation rates amongst the population with low SES might be due to the nature of tobacco control policies/interventions implemented in the country as associated with tobacco use.

During the Novel Coronavirus Pandemic, as part of a preventive measure, the National Directives for Covid-19 Management imposed a complete ban on all forms of tobacco [39]. More than 267 million individuals were unable to use tobacco due to its unavailability during the pandemic lockdown [40], and India might witness a low prevalence of tobacco consumption in the next years. The Global Youth Tobacco Survey (GYTS) [41] in 2019 reported a 42% decline in tobacco use among 13–15 years school going children in the last decade whereas the National Family Health Survey-5 [42] reported that 8.9% of women and 38% of men used tobacco in any form during 2029–202, an 43.9% and 18.9% reduction in tobacco use from the Global Adult Tobacco Survey 2016–2017.

### Strengths and limitations of the study

This study to the best of our knowledge is the first to provide insights into the socioeconomic differences in tobacco smoking and smokeless tobacco cessation among Indian men and women from 2009 to 2017 using nationally representative data. With 69,296 and 74,037 individuals above 15 years of age being interviewed during GATS 1 and GATS 2 to monitor and track key tobacco use and quit indicators, the results obtained were valid and robust. Despite the strengths of the study, a few limitations cannot be overlooked. The GATS data are cross-sectional self-reported surveys and therefore there might have been some bias in responses [3, 22]. Prevalence rates produced are representative at the national level and for key reporting domains: urban–rural divide, gender, and geographic regions of the country [43]. However, tobacco control efforts in India are planned by state governments and implemented at the district level, therefore aggregate regional estimates may not be adequate to plan, implement and monitor the impact of tobacco control interventions. Furthermore, when compared with the Census of India 2009 population projections, there is considerable under-sampling in the top 10 tobacco-consuming states [43] which may have led to an underestimation of estimates. Despite these limitations, the present study is reliable and sound to give a national overview of the socioeconomic differences in tobacco cessation and formulate policies accordingly.

## Conclusion

This study is the first to provide useful evidence on the relationship between tobacco cessation and socioeconomic characteristics among Indian men and women above 15 years of age. Tobacco cessation rates were observed to decline from 2009 to 2017 advocating the emergence of scale-up tobacco cessation services. Inequalities in tobacco cessation in India by wealth asset index and educational attainment suggest the need to formulate targeted interventions for different socioeconomic strata separately among men and women, as well as, separately for tobacco smoking and smokeless tobacco. This in turn will help to improve cessation rates to reduce tobacco consumption and associated premature morbidity and mortality.

## Supplementary Information

**Additional file 1: Table 1a.** Prevalence of tobacco smoking and smokeless tobacco and quit rates among Indian men above 15 years of age by wealth index during 2009-2010 and 2016-2017. **Table 1b.** Prevalence of tobacco smoking and smokeless tobacco and quit rates among Indian men above 15 years of age by educational attainment during 2009-2010 and 2016-2017. **Table 2a.** Prevalence of tobacco smoking and smokeless tobacco and quit rates among Indian women above 15 years of age by wealth index during 2009-2010 and 2016-2017. **Table 2b.** Prevalence of tobacco smoking and smokeless tobacco and quit rates among Indian women above 15 years of age by educational attainment during 2009-2010 and 2016-2017. **Table 3a.** State wise tobacco smoking cessation rates among Indian men above 15 years of age during 2009-2010 and 2016-2017. **Table 3b.** State wise smokeless tobacco cessation rates among Indian women above 15 years of age during 2009-2010 and 2016-2017.

## Data Availability

The datasets generated and/or analyzed during the current study are available from the World Health Organization South-East Asia Regional Microdata Repository (India—Global Adult Tobacco Survey 2009 (who. int) and India—Global Adult Tobacco Survey 2016–2017 (who. int)).

## Abbreviations

GATS: Global Adult Tobacco Survey
SES: Socioeconomic status
