# Supplementary material for: Socioeconomic inequalities in tobacco cessation among Indians above 15 years of age from 2009 to 2017: evidence from the Global Adult Tobacco Survey (GATS)
Source: BMC Public Health. 2022 Jul 26;22:1419. doi: 10.1186/s12889-022-13820-7 (PMC9321312; doi:10.1186/s12889-022-13820-7)
Supplement: Supplementary file 1 — Additional file 1: Table 1a. Prevalence of tobacco smoking and smokeless tobacco and quit rates among Indian men above 15 years of age by wealth index during 2009-2010 and 2016-2017. Table 1b. Prevalence of tobacco smoking and smokeless tobacco and quit rates among Indian men above 15 years of age by educational attainment during 2009-2010 and 2016-2017. Table 2a. Prevalence of tobacco smoking and smokeless tobacco and quit rates among Indian women above 15 years of age by wealth index during 2009-2010 and 2016-2017. Table 2b. Prevalence of tobacco smoking and smokeless tobacco and quit rates among Indian women above 15 years of age by educational attainment during 2009-2010 and 2016-2017. Table 3a. State wise tobacco smoking cessation rates among Indian men above 15 years of age during 2009-2010 and 2016-2017. Table 3b. State wise smokeless tobacco cessation rates among Indian women above 15 years of age during 2009-2010 and 2016-2017. [file 12889_2022_13820_MOESM1_ESM.docx]

**Supplementary File**

**Socioeconomic Inequalities in Tobacco Cessation among Indians above 15 years of age from 2009 to 2017: Evidence**

**from the Global Adult Tobacco Survey (GATS)**

Rufi Shaikh^a^, Dr. Nandita Saikia^b^

^a^Ph.D. researcher, International Institute for Population Sciences (IIPS),Mumbai, India (<https://orcid.org/0000-0003-2591-8873>)

^b^Professor at the Department of Public Health and Mortality Studies, International Institute for Population Sciences (IIPS), Mumbai, India (https://orcid.org/0000-0001-6735-6157)

**Corresponding author:** Rufi Shaikh

[Tel: +918108990229](Tel:+918108990229);

e-mail: rufi.shaikh95@yahoo.com

**Appendix Tables:**

Table 1a: Prevalence of tobacco smoking and smokeless tobacco and quit rates among Indian men above 15 years of age by wealth index during 2009-2010 and 2016-2017.

| **Tobacco Use** | | **Wealth Index (%)** | | | | | | | |
| --- | --- | --- | --- | --- | --- | --- | --- | --- | --- |
|  |  | **Low** | | **Medium** | | **High** | | **P value** | |
|  |  | **2009-10** | **2016-17** | **2009-10** | **2016-17** | **2009-10** | **2016-17** | **2009-10** | **2016-17** |
| **Tobacco Smoking** | Use | 23.46 | 18.24 | 28.87 | 25.23 | 32.96 | 29.88 | 0.000 | 0.000 |
|  | Cessation | 16.97 | 17.17 | 14.79 | 13.72 | 15.06 | 12.53 | 0.778 | 0.000 |
|  | Awareness | 90.86 | 98.27 | 82.05 | 95.37 | 59.56 | 84.06 | 0.000 | 0.000 |
|  | Perception | 92.84 | 99.17 | 92.45 | 98.63 | 89.16 | 97.22 | 0.000 | 0.000 |
| **Smokeless Tobacco** | Use | 20.81 | 17.24 | 33.13 | 32.12 | 45.79 | 45.73 | 0.000 | 0.000 |
|  | Cessation | 9.57 | 7.58 | 6.78 | 4.78 | 4.90 | 3.87 | 0.000 | 0.000 |
|  | Awareness | 87.33 | 97.43 | 87.30 | 93.04 | 75.50 | 82.36 | 0.000 | 0.000 |
|  | Perception | 92.20 | 98.90 | 90.95 | 98.58 | 85.98 | 97.81 | 0.000 | 0.000 |

Table 1b: Prevalence of tobacco smoking and smokeless tobacco and quit rates among Indian men above 15 years of age by educational attainment during 2009-2010 and 2016-2017.

| **Tobacco Use** | | **Educational Attainment (%)** | | | | | | | | | |
| --- | --- | --- | --- | --- | --- | --- | --- | --- | --- | --- | --- |
|  |  | **No formal Education** | | **Primary** | | **Secondary** | | **Higher and Above** | | **P Value** | |
|  |  | **2009-10** | **2016-17** | **2009-10** | **2016-17** | **2009-10** | **2016-17** | **2009-10** | **2016-17** | **2009-10** | **2016-17** |
| **Tobacco Smoking** | Use | 45.57 | 42.97 | 35.76 | 33.87 | 22.00 | 20.00 | 16.39 | 11.57 | 0.000 | 0.000 |
|  | Cessation | 13.77 | 13.28 | 13.38 | 13.58 | 18.61 | 14.43 | 18.73 | 15.63 | 0.093 | 0.780 |
|  | Awareness | 52.36 | 82.19 | 73.70 | 92.85 | 88.69 | 96.64 | 93.32 | 99.48 | 0.000 | 0.000 |
|  | Perception | 86.08 | 97.00 | 91.15 | 98.01 | 94.91 | 99.04 | 95.19 | 99.70 | 0.000 | 0.000 |
| **Smokeless Tobacco** | Use | 45.76 | 45.01 | 42.86 | 43.97 | 32.21 | 30.49 | 21.05 | 16.31 | 0.000 | 0.000 |
|  | Cessation | 5.44 | 3.94 | 5.43 | 4.51 | 7.43 | 5.11 | 6.48 | 6.17 | 0.010 | 0.563 |
|  | Awareness | 65.28 | 75.66 | 81.98 | 89.45 | 88.23 | 94.68 | 91.49 | 96.52 | 0.000 | 0.000 |
|  | Perception | 80.15 | 97.57 | 88.46 | 97.49 | 92.73 | 99.11 | 94.94 | 99.53 | 0.000 | 0.000 |

Table 2a: Prevalence of tobacco smoking and smokeless tobacco and quit rates among Indian women above 15 years of age by wealth index during 2009-2010 and 2016-2017.

| **Tobacco Use** | | **Wealth Index (%)** | | | | | | | |
| --- | --- | --- | --- | --- | --- | --- | --- | --- | --- |
|  |  | **Low** | | **Medium** | | **High** | | **P value** | |
|  |  | **2009-10** | **2016-17** | **2009-10** | **2016-17** | **2009-10** | **2016-17** | **2009-10** | **2016-17** |
| **Tobacco Smoking** | Use | 0.90 | 0.93 | 3.07 | 2.20 | 5.80 | 4.58 | 0.000 | 0.000 |
|  | Cessation | 9.56 | 17.34 | 17.87 | 12.98 | 20.17 | 14.66 | 0.486 | 0.148 |
|  | Awareness | 53.29 | 64.53 | 36.07 | 70.58 | 20.59 | 47.56 | 0.000 | 0.000 |
|  | Perception | 82.71 | 94.95 | 84.72 | 93.62 | 81.42 | 93.37 | 0.002 | 0.000 |
| **Smokeless Tobacco** | Use | 7.82 | 6.18 | 17.41 | 14.11 | 29.66 | 20.85 | 0.000 | 0.000 |
|  | Cessation | 7.91 | 9.31 | 6.68 | 7.01 | 7.19 | 5.45 | 0.500 | 0.000 |
|  | Awareness | 65.19 | 81.86 | 58.88 | 77.63 | 51.27 | 56.93 | 0.000 | 0.000 |
|  | Perception | 88.56 | 94.99 | 84.90 | 95.03 | 80.05 | 92.86 | 0.000 | 0.000 |

Table 2b: Prevalence of tobacco smoking and smokeless tobacco and quit rates among Indian women above 15 years of age by educational attainment during 2009-2010 and 2016-2017.

| **Tobacco Use** | | **Educational Attainment (%)** | | | | | | | | | |
| --- | --- | --- | --- | --- | --- | --- | --- | --- | --- | --- | --- |
|  |  | **No formal Education** | | **Primary** | | **Secondary** | | **Higher and Above** | | **P Value** | |
|  |  | **2009-10** | **2016-17** | **2009-10** | **2016-17** | **2009-10** | **2016-17** | **2009-10** | **2016-17** | **2009-10** | **2016-17** |
| **Tobacco Smoking** | Use | 7.44 | 6.01 | 1.67 | 1.44 | 0.30 | 0.43 | 0.14 | 0.23 | 0.000 | 0.000 |
|  | Cessation | 18.17 | 15.06 | 21.34 | 13.43 | 16.13 | 7.03 | 1.02 | 6.97 | 0.143 | 0.052 |
|  | Awareness | 22.66 | 51.79 | 54.93 | 80.20 | 76.79 | 82.08 | 96.68 | 99.37 | 0.000 | 0.000 |
|  | Negative Perception | 81.75 | 93.26 | 85.42 | 98.90 | 91.23 | 85.25 | 90.59 | 100.00 | 0.000 | 0.315 |
| **Smokeless Tobacco** | Use | 31.08 | 24.65 | 20.33 | 15.14 | 8.47 | 7.02 | 3.50 | 1.94 | 0.000 | 0.000 |
|  | Cessation | 6.23 | 6.47 | 6.95 | 7.34 | 10.69 | 4.89 | 18.77 | 10.79 | 0.015 | 0.129 |
|  | Awareness | 51.08 | 59.77 | 61.75 | 78.56 | 64.43 | 87.02 | 67.96 | 92.44 | 0.000 | 0.000 |
|  | Negative Perception | 78.49 | 91.82 | 88.63 | 96.55 | 88.76 | 99.26 | 96.90 | 99.54 | 0.000 | 0.000 |

Table 3a: State wise tobacco smoking cessation rates among Indian men above 15 years of age during 2009-2010 and 2016-2017

| **Geographic Region** | **STATE** | **Tobacco Smoking Cessation** | | | |
| --- | --- | --- | --- | --- | --- |
|  |  | **Men** | | **Women** | |
|  |  | **2009-2010** | **2016-2017** | **2009-2010** | **2016-2017** |
| **North** | Jammu & Kashmir | 16.29 | 6.81 | 21.86 | 11.86 |
|  | Himachal Pradesh | 21.24 | 9.77 | 39.66 | 0.00 |
|  | Punjab | 9.41 | 6.19 | 26.85 | 0.00 |
|  | Chandigarh | 4.53 | 12.41 | 8.69 | 0.00 |
|  | Uttarakhand | 17.18 | 11.51 | 22.47 | 13.93 |
|  | Haryana | 6.75 | 5.42 | 4.62 | 2.08 |
|  | Delhi | 3.37 | 13.07 | 35.51 | 16.37 |
|  | Rajasthan | 14.23 | 14.91 | 7.84 | 17.72 |
|  | Uttar Pradesh | 16.30 | 15.25 | 19.73 | 13.35 |
|  | Bihar | 31.43 | 31.52 | 36.53 | 16.27 |
| **NorthEast** | Sikkim | 9.89 | 6.17 | 11.52 | 4.82 |
|  | Arunachal Pradesh | 3.91 | 8.53 | 1.54 | 9.41 |
|  | Nagaland | 10.09 | 14.76 | 9.20 | 0.00 |
|  | Manipur | 10.33 | 10.08 | 10.61 | 19.14 |
|  | Mizoram | 6.70 | 6.12 | 6.47 | 11.58 |
|  | Tripura | 13.66 | 5.56 | 4.29 | 0.40 |
|  | Meghalaya | 4.17 | 4.43 | 0.00 | 2.87 |
|  | Assam | 5.84 | 17.14 | 4.98 | 52.64 |
| **East** | West Bengal | 3.58 | 8.08 | 2.40 | 2.74 |
|  | Jharkhand | 25.28 | 5.73 | 54.45 | 7.75 |
|  | Odisha | 11.93 | 21.82 | 0.00 | 69.18 |
| **Central** | Chhattisgarh | 16.74 | 18.14 | 4.61 | 60.85 |
|  | Madhya Pradesh | 14.89 | 12.95 | 9.17 | 2.81 |
| **West** | Gujarat | 19.43 | 8.11 | 20.24 | 32.76 |
|  | Maharashtra | 13.41 | 15.63 | 0.00 | 7.41 |
| **South** | Andhra Pradesh | 14.37 | 12.58 | 5.51 | 14.67 |
|  | Karnataka | 7.95 | 15.94 | 0.00 | 21.82 |
|  | Goa | 30.24 | 3.92 | 4.50 | 9.25 |
|  | Kerala | 33.15 | 29.36 | - | 37.24 |
|  | Tamil Nadu | 10.79 | 11.21 | 62.75 | 0.00 |
|  | Puducherry | 11.09 | 18.51 | 100.00 | 0.00 |
|  | Telangana | - | 12.14 | - | 12.57 |
| **India** |  | 15.35 | 13.98 | 18.90 | 14.39 |

Note: - State of Telangana was formed in 2014.

No female smoker in Kerala during 2009-2010, therefore no cessation

Table 3b: State wise smokeless tobacco cessation rates among Indian women above 15 years of age during 2009-2010 and 2016-2017

| **Geographic Region** | **STATE** | **Smokeless Tobacco Cessation** | | | |
| --- | --- | --- | --- | --- | --- |
|  |  | **Men** | | **Women** | |
|  |  | **2009-2010** | **2016-2017** | **2009-2010** | **2016-2017** |
| **North** | Jammu & Kashmir | 4.62 | 9.06 | 7.24 | 13.71 |
|  | Himachal Pradesh | 18.98 | 15.27 | 29.95 | 13.18 |
|  | Punjab | 3.86 | 1.71 | 0.00 | 0.00 |
|  | Chandigarh | 4.82 | 2.99 | 10.12 | 3.28 |
|  | Uttarakhand | 11.74 | 9.46 | 7.60 | 7.74 |
|  | Haryana | 3.22 | 4.71 | 6.79 | 0.00 |
|  | Delhi | 1.45 | 12.06 | 17.40 | 24.57 |
|  | Rajasthan | 9.53 | 7.68 | 9.10 | 13.66 |
|  | Uttar Pradesh | 5.64 | 4.33 | 6.88 | 8.32 |
|  | Bihar | 3.42 | 2.11 | 16.54 | 9.99 |
| **NorthEast** | Sikkim | 14.76 | 4.93 | 4.70 | 12.98 |
|  | Arunachal Pradesh | 3.15 | 2.44 | 2.78 | 3.49 |
|  | Nagaland | 8.47 | 4.39 | 14.94 | 2.95 |
|  | Manipur | 5.02 | 2.97 | 6.94 | 3.52 |
|  | Mizoram | 6.36 | 6.65 | 0.00 | 2.91 |
|  | Tripura | 7.00 | 0.77 | 3.52 | 1.12 |
|  | Meghalaya | 4.74 | 1.72 | 5.50 | 7.12 |
|  | Assam | 2.27 | 6.69 | 2.16 | 4.95 |
| **East** | West Bengal | 5.12 | 2.97 | 3.38 | 5.04 |
|  | Jharkhand | 4.08 | 1.93 | 15.38 | 4.61 |
|  | Odisha | 2.81 | 4.92 | 1.49 | 4.30 |
| **Central** | Chhattisgarh | 5.24 | 2.78 | 3.23 | 4.13 |
|  | Madhya Pradesh | 8.80 | 6.12 | 5.84 | 7.74 |
| **West** | Gujarat | 13.11 | 5.05 | 8.65 | 6.01 |
|  | Maharashtra | 3.95 | 3.60 | 3.73 | 5.81 |
| **South** | Andhra Pradesh | 10.55 | 10.34 | 1.37 | 1.89 |
|  | Karnataka | 4.73 | 7.75 | 8.66 | 7.01 |
|  | Goa | 7.64 | 2.31 | 26.45 | 13.74 |
|  | Kerala | 26.41 | 23.55 | 7.06 | 26.48 |
|  | Tamil Nadu | 5.64 | 4.45 | 3.42 | 5.66 |
|  | Puducherry | 18.83 | 5.97 | 9.86 | 16.32 |
|  | Telangana | - | 8.16 | - | 2.54 |
| **India** |  | 6.15 | 4.79 | 7.12 | 6.54 |

Note: State of Telangana was formed in 2014.
